# How the European Union legislations are tackling the burden of diabetes mellitus: A legal surveillance study

**DOI:** 10.3389/fpubh.2022.1002265

**Published:** 2022-11-23

**Authors:** Nour Mahrouseh, Szabolcs Lovas, Diana Wangeshi Njuguna, Noel Johny Nellamkuzhi, Carlos Alexandre Soares Andrade, Wilhelmina Egyirba Sackey, Anggi Septia Irawan, Orsolya Varga

**Affiliations:** ^1^Department of Public Health and Epidemiology, Faculty of Medicine, University of Debrecen, Debrecen, Hungary; ^2^Doctoral School of Health Sciences, University of Debrecen, Debrecen, Hungary; ^3^Faculty of Medicine, University of Debrecen, Debrecen, Hungary; ^4^Eötvös Loránd Research Network, Budapest, Hungary

**Keywords:** legal mapping, diabetes mellitus, obesity, non-communicable diseases, European Union

## Abstract

**Introduction:**

Surveillance of the European Union's (EU) legislations on the prevention of diabetes mellitus (DM) is needed, to more effectively tackle the rising prevalence of DM.

**Methods:**

This legal surveillance was carried out through a systematic search and screening, using EUR-Lex database to identify treaties, acts, and other legal documents for prevention of DM, non-communicable diseases (NCDs) and obesity, followed by their content analysis and assessment according to DM specific guidelines, target population and functional categories.

**Results and discussion:**

We found 22 legislations aimed at preventing DM, NCDs and obesity, but only 5 out of them specifically addressed preventing DM. The aims of legislations covered a broad spectrum of themes indicated by DM specific guidelines, mostly initiatives of life-course approach in preventing DM, NCDs and obesity from the area of energy intake. The target group of most legislations was the general population; high-risk subgroups such as pregnant women were hardly ever the primary target group. Our results prove that the EU has made cross-sectoral legislative efforts to reduce the disease burden and prevent DM but does not exhaust all possibilities. However, given its persistently rising DM prevalence, it is imperative to make sure that DM is a top health priority for various EU authorities and is incorporated into new initiatives, policies and laws.

## Introduction

Diabetes mellitus' (DM) prevalence in the European Union (EU) has increased from 6.63% since 2000 to 10.66% in 2019 (1) which is expected to continue rising in the upcoming decades (2–4). Such an increase has significant implications on premature mortality rates and quality of life, coupled with the expanding economic burden. In 2019, financial burden due to DM is estimated to be about 9% of the total healthcare expenditure in the EU (5, 6). This deteriorating trend is not inevitable. Type 2 diabetes mellitus (T2DM) as the most common type of DM which accounts for 90% of cases worldwide can be effectively prevented by addressing health determinants and risk factors (7).

Despite efforts to put DM high on the EU political agenda until 2012, it is now approached as part of non-communicable chronic diseases (NCDs). This approach is reasonable, as the most common NCDs, such as cardiovascular disease, cancers, asthma and other chronic obstructive pulmonary diseases, and DM are associated with the same risk factors. However, this common position masks differences between diseases, e.g., the different weight of risk factors in different diseases. Smoking is a risk factor for DM, but not as high a risk factor as obesity. Cigarette smokers have a higher risk of developing T2DM than non-smokers, at around 30–40% (8). Obesity is the most prominent T2DM risk factor (9), which leads to insulin resistance and disease development. Obese individuals are at an estimated higher risk of developing T2DM, around 80–85% (10) (11).

Legislation making in the field of NCDs' prevention is often considered less successful. For example, most obesity legislations of the past three decades failed to reduce obesity, for many reasons (12). One root problem is that these legislations focus on individuals, shifting the burden of actions onto them and ignoring the role of other factors, e.g., manufacturers and marketing companies (13). Another frequently mentioned problem is about the lack of proper enforcement and the ineffectiveness of self-regulatory and voluntary codes—such as rules restricting online advertising of unhealthy foods for children or refined sugar and salt in food—urging the need for stronger regulatory measures (14).

In the EU, the area of health, the member states have large autonomy and are almost entirely responsible for setting legislations and taking actions. However, beyond the member states, the EU itself has responsibility toward protecting and preserving the health of its population, through applying a wide and diverse set of legislations. This can be done *via* fiscal legislations, common market rules or public health legislations under the Treaty on the Functioning of the European Union (TFEU). According to the TFEU Article 168 on public health, a high level of human health protection shall be ensured in the definition and implementation of all Union legislations and activities. The EU treaties require the EU institutions to take health objectives and aspects into account in all legislation areas. Yet this obligation is at best, only rarely respected in many EU legislation areas (15).

In fact, the EU has no competence to adopt legally binding measures in the field of health, except in a few areas (e.g., measures setting high standards of quality and safety for medicinal products). EU soft law, although not legally binding, has a certain normative or coercive effect. This means that the EU mainly adopts soft laws that member states can choose whether or not to implement (16).

Regardless of the lack of a dedicated legal basis for harmonized prevention of DM, the EU may have the potential in improving population health and coordinating member states' health strategies. On several occasions, the Court of Justice of the European Union has ruled on how the EU can use e.g., Article 114 as a legal basis to pursue public health objectives through the integration of the internal market (17, 18). Very important examples are the Tobacco Products Directive (Directive 2014/40/EU; applicable from 2016) and the Tobacco Tax Directive (Council Directive 2011/64/EU) which have been landmarks in the fight against NCDs.

Legal and public health experts recognize law as one of the most important determinants of public health (19). Legal surveillance, including systematic, scientific collection and analysis of legislations relevant to public health, is critical to determine where we are and where we are heading under the current legal framework (20). Comparing different types of legislations offers a great opportunity to improve and raise the quality of underperforming preventive services.

This legal surveillance study was to map the EU legislations on DM prevention in order to get a coherent picture of legal efforts made by the EU.

## Materials and methods

The research is divided methodologically into two parts: identification of EU legislations, and their content analysis including assessment according to DM specific guidelines, target groups and functional categories.

### Identification of EU legislations

A systematic search was conducted by (NM and ASI) to detect and screen relevant legislations by using the EUR-Lex database (21). In our current use, the term 'legislation' is broad, covering treaties, legal acts and various types of soft laws. As the regulation of DM prevention overlaps to a large extent with the regulation of obesity and NCDs, the search was extended to these areas as well. The search was conducted in two rounds, one focused on DM and one involved obesity and NCDs. Search terms ”obes^*^ OR: non-communicable diseases” and “diabet^*^” were applied in title or text; the search language was English with no time limit. The search was restricted to treaties, legal acts, consolidated texts, international agreements, preparatory documents, European Free Trade Association (EFTA) documents. The EU treaties are binding agreements between EU member countries. The current treaty in force is the Treaty on the Functioning of the EU (TFEU). The main legal acts, based on the Treaties are regulations, directives, decisions, recommendations and opinions. Consolidation texts are the preliminary act and all its subsequent amendments of a single document. International agreements are agreements including member states, the EU and/or the European Atomic Energy Community with other countries or with international organizations. Preparatory documents are used in the process of preparing EU legislation, produced during the various stages of the legislative and budgetary process. The EFTA documents are by the EFTA institutions produced to promote free and economic trade between their members, within Europe, and globally (22).

**Figure 1:**
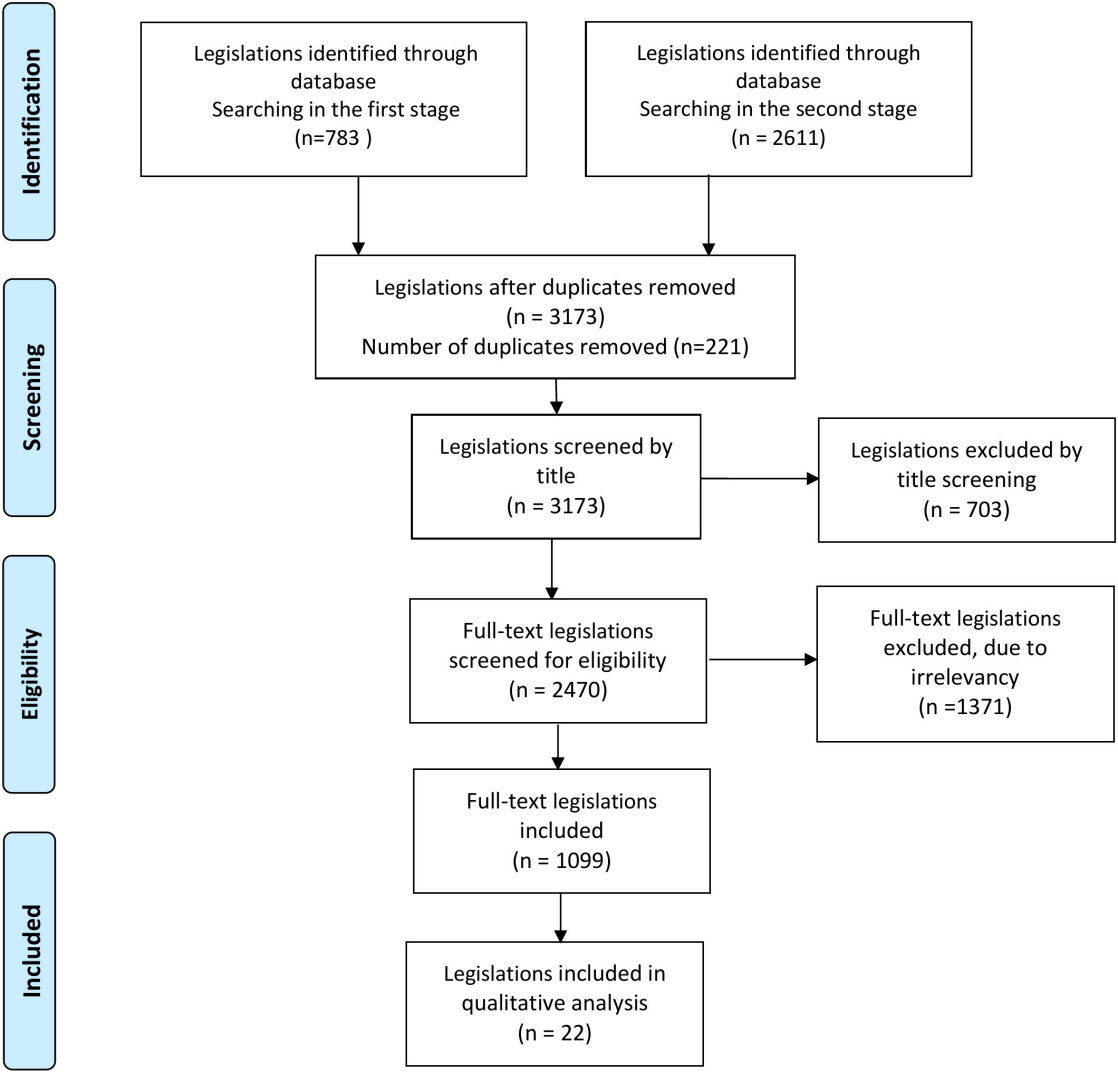
Selection process of EU legislations.

The screening process was carried out between October 2021 and January 2022 by (NM and ASI). After removal of duplicates (manually by using excel spreadsheet), legislations were manually screened for relevance; first phase of the selection was based on titles, then the second was based on full-text. The screening process is presented by Figure 1.

Legislations have been categorized as either indirect which address DM, NCDs or obesity in their text as part of the EU approach to health across all policies, or direct which address prevention of DM, NCDs or obesity in their text. All identified legislations were classified upon their EuroVoc terms and their authorship. EuroVoc is a multilingual vocabulary list, generated for documentary information produced by EU institutions, comprising different topics and domains, covering the activities of the EU. It enables researchers to conduct systematic searches using a controlled vocabulary and carrying out semantic networks between concepts. It is divided into 21 domains and numerous subdomains (23).

### Content analysis

Only legislations specifically adopted for the prevention of DM, NCDs or obesity directly were subject to content analysis. The content analysis of identified specific documents were carried out *via* the MonQcle, the Legal Text Document Analysis Platform (24). Each document as a record was added to MonQcle database and coded by two coders (NM, ASI), independently. After the initial review of the legislations had been completed, a list of themes on which each document should be coded was defined. This original list of themes addressing the goals and methods partly emerged from the WHO document entitled Global report on diabetes (25) and the Joint paper of the European Public Health Alliance (EPHA) “Toward an EU strategic framework for the prevention of NCDs” (26).

The themes derived from the WHO Global report on diabetes effective legislation options were: a life-course approach to preventing diabetes, improving early childhood nutrition, supportive environments for physical activity, settings-based interventions, fiscal, legislative and regulatory measures for healthy diet, trade and agricultural policies that promote healthy diets, regulation of marketing of food's high in sugars, fats and salt, education, social marketing, mobilization and preventing diabetes in people at high risk (25).

The themes derived from the EPHA Joint paper “Toward an EU strategic framework for the prevention of NCDs strategic priorities and specific actions” were: implement the WHO “Best buys,” tackle health inequalities, adopt a rights-based approach, elaborate a pan-European system for data collection, policy evaluation and accountability, ensure inter-institutional coordination on health and well-being and a policy home for health within the European Commission structure, launch a “Health in All Policies” online policy portal and pursue “EU flagship initiatives” in areas that can deliver co-benefits for NCD prevention and other Sustainable Development Goals (SDGs) (26).

The original themes of the variables from the guidelines were reviewed several times and new themes were added; with each change, the legislations were reviewed again to ensure that all available data were coded. Themes also covered legislation enforcement types: binding (e.g., regulations), non-binding (e.g., white papers) and conditionally binding (e.g., decisions). In order to understand better the themes of the guidelines and in line with the modified classification of Timpel et al. the theme of target groups of legislations were coded as pregnant women and young families, children and adolescents, working age population, the elderly, general population, governments, communities or non-governmental organizations (NGOs), and not defined or not applicable (27). Functional categories used by legislations to address the following determinants of DM, NCDs, and obesity were coded as themes that included energy intake, energy expenditure, provision of information, screening and treatment (28). The validity (when applicable) and legislator/author of the documents are recorded based on the original data extracted from Eur-Lex. The final list of themes (see Supplementary Table S1) was used as categorical variables in the analysis.

### Statistical analysis and internal validity

Descriptive analyses were used to describe the frequency of legislations by themes derived from the guidelines; WHO Global report on diabetes and EU strategic framework for the prevention of NCDs, target groups and functional categories. The visualization of the analysis was carried out with the help of the following programs: Gephi 0.9.5 to produce a cluster network analysis, TIBCO Cloud Spotfire analyst to produce a heatmap.

The quality appraisal (internal validity) consisted of evaluating each legislation document, based on similarity of coding by two independent reviewers (NM, ASI), systematically. Conflicts were solved in open discussion, involving an independent reviewer (OV). Inter-rater reliability for each legislation was assessed using Cohen's kappa (k) inter rater reliability test and its categorization (29).

## Results

### Description of the identified legislations

The data collection resulted in 1099 relevant legislations on DM, NCDs and obesity, and of these 1099 legislations, 22 were specifically aimed at the direct prevention of DM, NCDs and obesity. The five legislations specifically aimed at preventing DM are: Addressing the EU diabetes epidemic European Parliament resolution of 14 March 2012 on addressing the EU diabetes epidemic [2011/2911(RSP)], Commission staff working document—Summary of dietary recommendations for people with diabetes, Commission staff working document—Summary of main points of scientific basis of the dietary recommendation for diabetics, Council conclusions on promotion of healthy lifestyles and prevention of Type 2 diabetes and Report from the Commission to the European Parliament and the Council on foods for persons suffering from carbohydrate metabolism disorders (diabetes).

**Figure 2:**
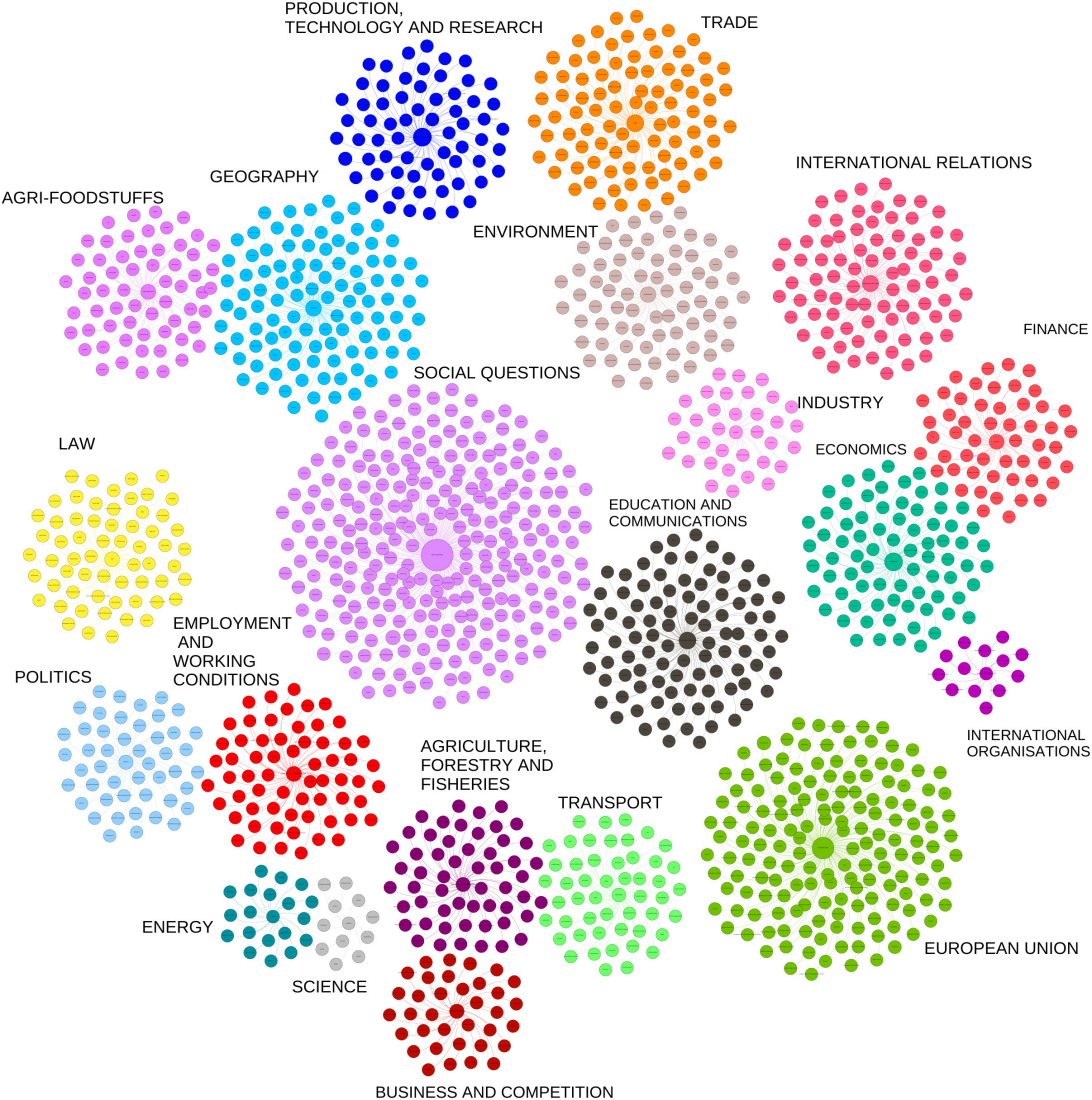
Cluster structure of EuroVoc classifications. Legend, EuroVoc terms are represented by a network analysis in form of clusters. Each cluster represents a EuroVoc domain. In the center of the cluster the domain node is located, and each branch (edge) represents a subdomain. The size of the node depends on how often the term is used in the legislations.

**Figure 3:**
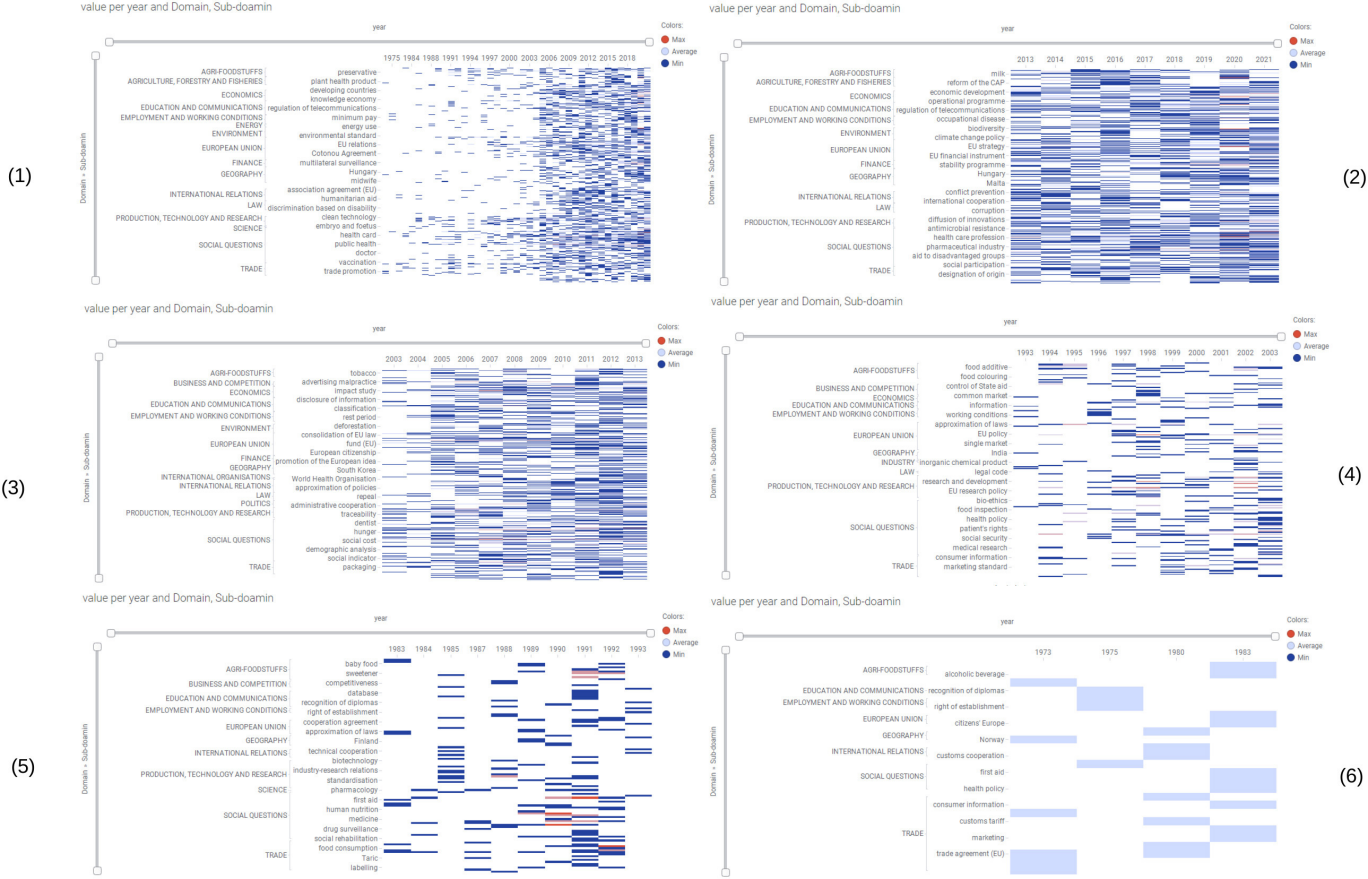
Snapshots of the interactive heatmap of the most commonly used EuroVoc terms over the years. Legend, The heatmap contains domains and subdomains in the columns and years in the rows. The interactive heatmap allows users filtering by years, domains and subdomains. Snapshot (1) represents the heatmap covering EuroVoc terms from 1973 to 2021, (2) from 2013 to 2021, (3) from 2003 to 2013, (4) from 1993 to 2003, (5) from 1983 to 1993 and (6) from 1973 to 1983. The colors indicate the number of EuroVoc terms. The interactive heatmap is available at the following link: https://eu.spotfire-next.cloud.tibco.com/spotfire/wp/OpenAnalysis?file=4499a74c-a743-4844-bde1-81b018f81f96.

The identified legislations covered the period from 1968 to 2022. Of these, 366 are legislations that are currently in force. Only 853 legislations had EuroVoc terms classifications. A total of 6189 EuroVoc terms belonged to 853 legislations, 1486 unique EuroVoc terms were identified, as one EuroVoc term appeared in several legislations. Figure 2 shows the distribution of EuroVoc terms indicating that the most prevalent EuroVoc domain is the social questions covering family, health, social framework, social affairs and protection, culture and religion, construction and town planning subdomains. However the subdomain with most records was the public health. Figure 3 shows the distribution of EuroVoc terms over time, indicating how the regulatory emphasis has changed over the years. The number of legislations has increased significantly since year 2005. While in 1973 the most common subdomains focused on trade as custom duties, trade agreements, etc.; in 2021 the most common subdomains were really diverse and focused on areas related social questions as epidemic, corona virus, disease prevention, etc., in addition to other domains as economics and European Union. An interactive heatmap of the most commonly used EuroVoc terms over the years was generated which is available at the link: https://eu.spotfire-next.cloud.tibco.com/spotfire/wp/OpenAnalysis?file=4499a74c-a743-4844-bde1-81b018f81f96; its filtering by year, domains or sub-domains is possible.

### Content analysis

Legislations that addressed prevention of DM, NCDs or obesity included 6 binding legislations-−5 regulations and one conditional binding decision—and 17 non-binding legislations, see Table 1. The entire list of documents is presented in the Supplementary Table S1, along with their validity period.

**Table 1:** Assessment of legislations according to the WHO Global report on diabetes and EU strategic framework for the prevention of NCDs guidelines.

**Themes**	**Binding**	**Non-binding**	**Total**
A life-course approach to preventing diabetes	2	18	20
Improving early childhood nutrition	1	11	12
Supportive environments for physical activity	0	11	11
Settings-based interventions	1	9	10
Fiscal, legislative and regulatory measures for healthy diet	2	12	14
Trade and agricultural policies that promote healthy diets	3	11	14
Regulation of marketing of foods high in sugars, fats and salt.	3	11	14
Education, social marketing and mobilization	2	12	14
Preventing diabetes in people at high risk	0	12	12
Implement the WHO “Best buys”	2	11	13
Tackle health inequalities and Adopt a rights-based approach	0	4	4
Elaborate a pan-European system for data collection, policy evaluation and accountability	0	3	3
Ensure inter-institutional coordination on health and well-being and a policy home for health within the European Commission structure	0	5	5
Launch a “Health in All Policies” online policy portal	0	0	0
Pursue “EU flagship initiatives” in areas that can deliver co-benefits for NCD prevention and other SDGs*	1	7	8

Legend, During coding each legislation might have fallen under several categories. WHO: World Health Organization, NCDs: Non-communicable diseases. Sources of the variables shown in the first column are according to the WHO documents entitled Global report on diabetes (25) and the joint paper of the European Public Health Alliance (EPHA) “Toward an EU strategic framework for the prevention of NCDs” (26).

The authors of the legislations varied, with one or more EU institutions identified as legislators. The European Commission has produced most legislations with 13 legal documents (see Supplementary Table S1).

#### Assessment of legislations according to the WHO global report on diabetes and EU strategic framework for the prevention of NCDs guidelines

The most prevalent classification category was a life-course approach to preventing DM (*n* = 20), including mostly non-binding legislations. The least addressed theme was the Elaborate a pan-European system for data collection, legislation evaluation and accountability. The theme of Launch a “Health in All Policies” online legislation portal was not addressed by all EU legislations at all. No mandatory legislation available for 6 themes. The most prevalent binding legislations were food related including trade and agricultural laws that promote healthy diets (*n* = 3) and regulation of marketing of foods high in sugars, fats and salt (*n* = 3).

#### Assessment according to target groups and functional categories

The target groups varied according to the legislation, with most of the legislations targeting the general public or communities, see Table 2. Only some non-binding legislations addressed pregnant women, organizations or the countries (governments). Furthermore, even non-binding legislation does not target governments.

**Table 2:** Target groups of legislations, by the type of legislation.

**Themes**	**Binding**	**Non-binding**	**Total**
General	3	15	18
Children	1	12	13
Pregnant women		1	1
Organizations		6	6
Community	2	14	16
Countries		7	7

Legend, During coding each legislation might have fallen under several categories.

The functional categories used in legislations covered largely the area of energy intakes, see Table 3. In our dataset, food labeling and the provision of healthy food were part of food intake mechanism used to directly target DM, NCDs and obesity. Legislations with energy expenditure mechanism dealt with physical activities such as community programs or constructing necessary infrastructure. Information legislations provide measures for health promotion, education and research and innovation. At least in part, EU strategies incorporated screening and treatment legislations linked to detection, early diagnosis and treatment, and improving patients' quality of life. Non-binding legislation exists exclusively in the field of energy expenditure and screening and treatment.

**Table 3:** Functional categories of legislations, by the type of legislation.

**Themes**	**Binding**	**Non-binding**	**Total**
Energy intake	3	16	19
Energy expenditure		13	13
Information	2	11	13
Screening and treatment		9	9

Legend, During coding each legislation might have fallen under several categories.

### Internal validity

Coding similarity of main themes was assessed by indicating the level of agreement/disagreement between the two raters. Results of Cohen's kappa of the WHO Global report on diabetes and EU strategic framework for the prevention of NCDs and functional categories were interpreted as excellent agreement 0.805 and almost perfect agreement, 0.945, between the two coders (NM,ASI); level of agreement for the target group was interpreted as substantial agreement, 0.788.

## Discussion

The aim of the article was to provide a systematic overview of legislations contributing to prevention of DM in the EU. To the best of our knowledge, this is the first paper analyzing EU efforts and legislative gaps in the field of DM prevention.

### Prevention of DM is not at the heart of legislation

Our key finding shows that several legislations covered the prevention of DM as part of NCDs or *via* risk factors. However, only very few non-binding legislations addressed DM, specifically between 2006 and 2012. The Council concluded the Austrian conference on “Prevention of type 2 diabetes” by stressing the importance of promoting healthy lifestyles and preventing T2DM, given that DM is a major cause of premature death and mortality and a factor affecting the quality of life of EU citizens (30). This was followed by a Commission report on provision of foods for persons suffering from DM that included dietary recommendations for people with DM and information on the current EU legislation of these foods and proceeded by other dietary recommendations (31–33). In 2012, the European Parliament published a resolution addressing DM epidemic and calls upon on placing DM high on EU agenda (34). Despite this, DM never became front and center in the legislative process. However, a new initiative called “The Blueprint for Action on Diabetes in the European Union by 2030“ calls for DM to be brought to the center of the legislative agenda (35) which may lead to DM-specific EU soft laws in the near future.

Since their launch, the health programs have been aimed at fighting diseases and improving the health of EU citizens through projects and grants. DM is a target disease for health programs, as it can be prevented as an NCD by changing risk factors such as obesity and sedentary lifestyle, and by providing medication to prevent it. The first health programs were effective and had a significant impact on national health systems (36, 37). The current health program is EU4health, which has received more funding than previous programs because of the greater focus on health in the allocation of the financial budget (38). The recent COVID pandemic has hit the disadvantaged and the elderly hardest, largely due to high levels of obesity and DM; underlining the need for future programs to place greater emphasis on their prevention (39).

### Shifting legislative focus

The strategies of EU targeting NCDs including DM have changed through time. Early legislations focused more on the EU market regulations. The EU common market allows and regulates the movement of goods including food products and the set custom tariffs for these products between the EU and other countries (40). These legislations regulate the food and changes in the food market and decide upon the availability of products that could be purchased by EU citizens. The EuroVoc terms have changed since 2010 to encompass more preventive approaches to NCD risk factors. Risk factors are the cornerstone of preventive health legislations, which is presented by high number of EuroVoc terms concentrated on obesity, health programs, etc. Another frequent sort of legislation provides information on health by setting out guidelines and informing the lay public on illness management, symptoms, and risk factors and ensure the right to health. In 1993, the Commission published a communication on a framework for action in the field of public health, laying down eight areas for action, including health promotion, and this served as the model for future public health programs (41).

### Interpretation of the content analysis

Most of the legislations fell under a life course approach strategy that was suggested by the WHO diabetes report. This approach has proven its efficacy through minimizing risk factors and enhancing protective behaviors and factors through important phases of individuals, from the perinatal period through childhood and adolescence, to adult life. The trade, agriculture and fiscal legislations to improve healthy diet were also part of EU strategies. The single market approach of the EU has a spill-over effect on health. Public health is directly financed by a few instruments in the EU including health programs funds (36, 37); and DM was on the agenda of health programs as written above. Informational legislations include health promotion, education, and research and innovation, which have recently been among the EU's priorities and have been the subject of significant spending (42).

Food taxation, as type of fiscal legislations were not applied on the EU level, unfortunately, taxation is applied as a voluntary measure in some states, exclusively (43). One example of current taxation is the tax on sugar-sweetened beverages (SSBs), introduced in France in 2011 (44). A Mexican study reported that SSBs taxation has reduced consumption (45), especially in the lower classes of society, thus may result in reducing DM disease burden. Sugar legislations have yielded similar results in other context as well (43). The implementation of such legislations should be encouraged in the member states.

Legislation mechanism as the aspect of targeting energy intake, that included promoting, education and regulating the marketing of foods high in sugars, fats and salt was a part of the EU approach through labeling and media advertisement to subsiding with healthier options. A systematic review' results stating consumer information through labeling is the major form of nutritional legislations in the EU (46). Food labeling has been proven to be effective in many countries (47), as confirmed by a meta-analysis on food labeling reporting that providing food labels and nutritional claims can increase healthy consumption (48) by their effects on consumers choices (49), justifying a focus on food information and labeling in EU legislations. Food environment plays an essential role in defining food choices. Wide range of evidences around the globe have confirmed the availability of healthy food and food for intended purposes can improve the life quality of a population and their eating habits, thus prevent NCDs. Improving diet at an early age can likely to prevent the development of obesity and DM later in life (50).

On the other hand, the issue of an environment conducive to physical activity should also be at the heart of EU legislation, as reflected in the functional category of energy use, which included legislations on physical activity and its structure. Studies have found that the effectiveness of physical activity and sedentary behaviors legislations worldwide ranges from low to moderate (51). Considering that physical activity is the most important preventive factor against DM, physical activity legislations need to be modified if they have proved ineffective (51). In the EU, legislation on physical activity is the responsibility of the member states, with the Commission only supporting and coordinating national measures.

There is a consistent and profound health gradient in Europe between socio-economic groups. Social legislations may influence health indirectly by targeting socioeconomic factors (52). Among the identified legislations, the White Paper on a Strategy for Europe on Nutrition, Overweight and Obesity related health issues had discussed the importance of targeting socioeconomic factors and reducing inequality in order to lessen obesity and other NCDs as DM in the EU (53). Targeting vulnerable groups may reduce the social gradient and help in preventing DM.

The most prevalent target group in the legal documents was the general population. The numbers alone show that the structure of the legislation is not sophisticated, no specific attention is paid to subgroups, especially pregnant women. Legislations specifically tailored to children, such as the “Council conclusions to contribute toward halting the rise in Childhood Overweight and Obesity,” can be an effective approach to preventing DM by targeting individuals at high risk to prevent the early onset of DM by shaping the associated behaviors of the individual (54).

Screen and treat legislations were partly in the focus of the EU strategies, ranging from the detection, early diagnosis and treatment as well as improving the quality of life for patients. More than 38% of DM patients in the EU go undiagnosed and many have already had one or more complications by the time of diagnosis (35). In addition to being extremely costly for health systems, these problems have terrible human consequences. The document Blueprint for Action on Diabetes in the European Union by 2030 has suggested to develop an EU-wide screening tool and framework for people at risk to be applicable by the year 2024; early interventions should be consistent, sustainable and start as early as possible, in a supportive environment, targeting risk factors (35).

Although no legislation was identified under the theme “Launch a ‘Health in All Policies' online policy portal,” several stakeholders called for its launch. Such an online policy portal could provide transparency on related health legislations and instruments and help or guide the application of all health policies, as well as enable online discussion and collaboration between health and other stakeholders. This could also help to achieve the other goal of ensuring inter-institutional coordination on health and well-being, and to give health a political home within the European Commission. The EU already has recently established a new interactive tool to collect legislations under the fund of EU4Health “EU Health Policy Platform,” which may be a step toward forming the Health in All Policies' online policy (55, 56).

In summary, there is limited binding legislation on the prevention of DM. Although the number of relevant soft laws is quite high, the EU does not make use of the possibilities available, for example there is no ”Health in All Policies“ online policy to monitor the impact of EU legislation. All DM-specific legislations are at least 10 years of non-binding soft laws. The EU has started to focus on risk factors, but mostly through energy intake mechanism, rather than covering the full spectrum of mechanisms for prevention. Little attention is paid to high-risk groups that could face serious consequences in the event of a future outbreak.

An important question is: how will EU legislation on diabetes prevention and control change? As described above, today's public health challenges cannot be effectively tackled within the framework of nation states, but under the TFEU, health policy is essentially a national competence, and the 27 member states have long been adamant that this should remain the case. Building on the experience of COVID-19, the European Commission announced in autumn 2020 a vision and a package of actions for a European Health Union with the long-term aim of developing common, or at least shared, health competences between member states and the EU in a number of areas (57). The report of a series of civil consultations on the future of Europe, which ended in 2022, no longer ruled out amending the EU's founding documents in this direction (58). Achieving this will be a longer process, but guaranteeing the quality of life and health security of EU citizens will not allow EU health policy and health law to operate only with soft instruments in the future.

### Limitations

Although not all types of DM is considered preventable, legislations may not distinguish DM subtypes but regulate the field as an overall DM category.

While this qualitative research presents novel findings on the implementation of the WHO Global report on diabetes and EU strategic framework for the prevention of NCDs, legal mapping has limitations. There are concerns that existing EU legislations do not reflect actual implementation in the member states. There is a gap between tracking and achieving what is proposed and launched, and the extent to which it is implemented. Furthermore, EU legislations might be effective, but the impact of legislation changes may take years to materialize. Finally, it is unclear from this analysis whether EU legislations are effective in preventing, detecting and responding to disease, and further 'legal epidemiology' research will be needed to study the impact of legislations on national health systems and public health outcomes based on these findings (59).

## Conclusions

Legislations targeting DM prevention are limited in terms of number and scope. The EU has made significant efforts to legislate against DM, NCDs and obesity since the 1960s, but the prevalence of DM is still increasing in most EU member states. Such increase could be attributed to physical inactivity and dependence on western diet, socioeconomic characteristics and their interactions with an aging population (5). As DM is a complex disease, the success of legislations requires a multiplicity of political forces. It is critical to ensure that DM is high on the agenda of various EU institutions, to ensure DM and its risks factors are included in all new relevant initiatives, programs and legislations. We believe that a more comprehensive and ambitious legislative network proposed by the WHO could be adopted in the EU.

## Data availability statement

The original contributions presented in the study are included in the article/Supplementary material, further inquiries can be directed to the corresponding author.

## Author contributions

Conceptualization: NM and OV. Data curation and visualization: NM. Formal analysis: NM, AI, and OV. Project administration: DN, CS, and OV. Supervision and funding acquisition: OV. Methodology, writing—original draft, and writing—review and editing: NM, SL, DN, NN, CS, WS, and OV. All authors contributed to the article and approved the submitted version.

## Funding

DN and CS are supported by the Stipendium Hungaricum Scholarship. OV receives fellowship by the Hungarian Academy of Sciences (Premium Postdoctoral Research Program) grant number 3134/2019/KP. Project no. FI17198 has been implemented with the support provided by the National Research, Development, and Innovation Fund of Hungary, financed under the FK_22 funding scheme.

## Conflict of interest

The authors declare that the research was conducted in the absence of any commercial or financial relationships that could be construed as a potential conflict of interest.

## Publisher's note

All claims expressed in this article are solely those of the authors and do not necessarily represent those of their affiliated organizations, or those of the publisher, the editors and the reviewers. Any product that may be evaluated in this article, or claim that may be made by its manufacturer, is not guaranteed or endorsed by the publisher.
