# Supplementary material for: How the European Union legislations are tackling the burden of diabetes mellitus: A legal surveillance study
Source: Front Public Health. 2022 Nov 23;10:1002265. doi: 10.3389/fpubh.2022.1002265 (PMC9727193; doi:10.3389/fpubh.2022.1002265)
Supplement: Supplementary file 1 [file Table_1.DOCX]

Supplementary Material

# Overview of the themes

## Themes of the WHO Global report on diabetes and EU strategic framework for the prevention of NCDs guidelines:

A life-course approach to preventing diabetes: Legislative actions that ensure prevention of DM through a life-course view as early childhood is a crucial time for intervention to reduce the risk of obesity and diabetes later in life since, when food and exercise habits are formed and when the long-term management of energy balance may be set. This approach also acknowledges the rising risk that comes with diabetes would also be by a life-course strategy. (1)

Improving early childhood nutrition: Strategies to enhance early childhood nutrition are important, which include steps that need to be taken with the improvement of maternal health, baby and early child nutrition from a woman's pregnancy through early stages of childhood to foster physical activity, reduce the use of foods heavy in calories, fats, sweets, and salt, and encourage growth. (1)

Supportive environments for physical activity: Actions to reduce overweight and obesity are crucial to avoiding DM, and urban planning and active transport regulations may guarantee that walking, cycling, and other non-motorized modes of transportation are available. Additionally, the physical environment may offer sporting, recreational, and entertainment opportunities and guarantee that both kids and adults have enough safe places to engage in active life. (1)

Settings-based interventions: Interventions focused on the environment can help in DM prevention and management. The goal of these treatments is to execute both population-wide and individualized high-risk interventions inside families and communities where people reside, learn, work, and play. (1)

Fiscal, legislative and regulatory measures for healthy diet: Legislative actions to increase the price of foods high in fat, sugar and salt which can lead to decrease in their consumption. Likewise, a price reduction of healthy food can help consumers in making better choices. (1)

Trade and agricultural policies that promote healthy diets: Legislations include boosting the production of fruits and vegetables and improving diet through facilitating sustained long-term production, transport and marketing of healthier foods. (1)

Regulation of marketing of foods high in sugars, fats and salt: Legislations that include actions that control the marketing of food to minors, can direct customers toward better food selections through nutrition labelling, calorie count, etc. (1)

Education, social marketing and mobilization: Legislative actions of ongoing media efforts and educational initiatives aimed at promoting healthy eating (or discouraging the intake of unhealthy foods) and encouraging physical exercise. (1)

Preventing diabetes in people at high risk: Legislations of concentrated interventions to change people’s diet, increase physical activity and loss of body weight for people with impaired glucose tolerance, with or without impaired fasting glucose. (1)

Implement the WHO ‘Best buys’: Legislations that support the WHO’s "Best Buys and other recommended, interventions" which are a collection of the most promising policy types, evaluated for cost-effectiveness or recommended based on other evidence that addresses the drivers of NCDs. The "best buys" provide actions for four major NCD risk factors, including smoking, alcohol use, poor diet, and insufficient physical activity, as well as for four disease categories, including cardiovascular disease, diabetes, cancer, and chronic respiratory disease. (2)

Tackle health inequalities and adopt a rights-based approach: Legislations that target health inequalities through socioeconomic factors and the poorest population and other disadvantaged groups. (2)

Elaborate a pan-European system for data collection, policy evaluation and accountability: Legislations that host an EU-wide health data system with registries for NCD incidence, prevalence, health outcomes, costs, and key risk factor indicators, address gaps in the data that is currently available and the methods used to collect it, offer to fund for data collection, and address the methods and data gaps. (2)

Ensure inter-institutional coordination on health and well-being and a policy home for health within the European Commission structure: Legislations that ensure inter- and intra-institutional coordination through this position, and co-ownership of cross-cutting policy files across the fields of public health and other areas. (2)

Launch a ‘Health in All Policies’ online policy portal: Legislations that establish a ‘Health in All Policies’ platform that may have a substantial influence on health and well-being, particularly NCDs, through a tracking tool and to be a repository for all published impact assessments of the policy initiatives. (2)

Pursue ‘EU flagship initiatives’ in areas that can deliver co-benefits for NCD prevention and other SDGs (Sustainable Development Goals): Legislative action that should be built around talks with organizations and professionals, and legislators with paying careful attention to how to handle any possible conflicts of interest and the effectiveness of the use of policy resources. (2)

## Themes of the functional categories:

Energy intake: Legislations of a healthy diet that may include laws of nutrition, labelling, calorie intake, food taxation, etc. (3)

Energy expenditure: Legislations of promoting physical activity that may include sport, structure, transportation, etc. (3)

Information: Legislations of data collection, health education, information about eating habits and exercise routines that support keeping a healthy weight, etc. (3)

Screening and treatment: Legislations of screening population, monitor high-risk patients and regulation of medicine. (3)

# Supplementary data for legislations

Table S1. List of legislations and their details including their CELEX numbers, dates of documents, dates of publication, dates of end of validity and authors.

| Title | CELEX number | Date of document | Date of publication | Author | Date of end of validity |
| --- | --- | --- | --- | --- | --- |
| Addressing the EU diabetes epidemic European Parliament resolution of 14 March 2012 on addressing the EU diabetes epidemic (2011/2911(RSP)) | 52012IP0082 | 3/14/2012 | 8/31/2013 | European Parliament | No end date |
| Commission staff working document - Summary of dietary recommendations for people with diabetes | 52008SC2295 | 7/10/2008 |  | European Commission | No end date |
| Commission staff working document - Summary of main points of scientific basis of the dietary recommendation for diabetics | 52008SC2294 | 7/10/2008 |  | European Commission | No end date |
| Council conclusions on promotion of healthy lifestyles and prevention of Type 2 diabetes | 52006XG0623(01) | 6/23/2006 | 6/23/2006 | Council of the European Union | |
| Report from the Commission to the European Parliament and the Council on foods for persons suffering from carbohydrate metabolism disorders (diabetes) | 52008DC0392 | 6/26/2008 |  | European Commission | No end date |
| 98/251/EC: Commission Decision of 21 May 1997 on the proposal of Austria to award aid to the Hoffmann-La Roche company for the development of the drug 'Orlistat', designed for the treatment of pathological obesity (Only the German text is authentic) (Text with EEA relevance) | 31998D0251 | 5/21/1997 | 4/3/1998 | European Commission | No end date |
| Commission Decision of 17 July 2018 setting up a Commission expert group ‘Steering Group on Health Promotion, Disease Prevention and Management of Non-Communicable Diseases’ and repealing the Decision setting up a Commission expert group on rare diseases and the Decision establishing a Commission expert group on Cancer Control (Text with EEA relevance.) | 32018D0718(02) | 7/17/2018 | 7/18/2018 | Directorate-General for Health and Food Safety, European Commission | 12/31/2023 |
| Commission Delegated Regulation (EU) 2017/1522 of 2 June 2017 supplementing Regulation (EU) No 609/2013 of the European Parliament and of the Council as regards the specific compositional and information requirements for total diet replacement for weight control (Text with EEA relevance) | 32017R1522 | 6/2/2017 | 9/6/2017 | European Commission, Directorate-General for Health and Food Safety | 9/6/2017 |
| Commission Delegated Regulation (EU) 2017/1798 of 2 June 2017 supplementing Regulation (EU) No 609/2013 of the European Parliament and of the Council as regards the specific compositional and information requirements for total diet replacement for weight control (Text with EEA relevance. ) | 32017R1798 | 6/2/2017 | 10/7/2017 | Directorate-General for Health and Food Safety, European Commission | No end date |
| Commission staff working document - accompanying the White paper on a Strategy for Europe on Nutrition, Overweight and Obesity related health issues - Summary of the Impact Assessment {COM(2007) 279 final} {SEC(2007) 706} | 52007SC0707 | 5/31/2007 |  | European Commission | No end date |
| Commission staff working document accompanying the White Paper on a Strategy for Europe on Nutrition, Overweight and Obesity related health issues Impact assessment {COM(2007) 279 final} {SEC(2007) 707} | 52007SC0706 | 5/31/2007 |  | European Commission | No end date |
| Council Conclusions of 2 December 2002 on obesity | 52003XG0117(01) | 12/2/2002 | 1/17/2003 | Council of the European Union | No end date |
| Council conclusions to contribute towards halting the rise in Childhood Overweight and Obesity | 52017XG0629(01) | 6/29/2017 | 6/29/2017 | Council of the European Union | |
| EU position and commitment in advance of the UN high-level meeting on the prevention and control of non-communicable diseases European Parliament resolution of 15 September 2011 on European Union position and commitment in advance to the UN high-level meeting on the prevention and control of non-communicable diseases | 52011IP0390 | 9/15/2011 | 2/22/2013 | European Parliament | |
| European Parliament resolution on 'Promoting healthy diets and physical activity: a European dimension for the prevention of overweight, obesity and chronic diseases' (2006/2231(INI)) | 52007IP0019 | 2/1/2007 | 10/25/2007 | Committee on the Environment, Public Health and Food Safety, European Parliament | |
| Green Paper - \Promoting healthy diets and physical activity : a European dimension for the prevention of overweight | /* COM/2005/0637 final */ | 12/8/2005 | 12/8/2005 | European Commission | No end date |
| Opinion of the Commission for Sustainable Development on A strategy for Europe on nutrition, overweight and obesity related health issues | 52007AR0312 | 2/7/2008 | 4/25/2008 | European Committee of the Regions | No end date |
| Opinion of the European Economic and Social Committee on Obesity in Europe — role and responsibilities of civil society partners | 52005IE1070 | 9/28/2005 | 1/31/2006 | European Economic and Social Committee | No end date |
| Position (EU) No 3/2013 of the Council at first reading with a view to the adoption of a Regulation of the European Parliament and of the Council on food intended for infants and young children, food for special medical purposes, and total diet replacement for weight control and repealing Council Directive 92/52/EEC, Commission Directives 96/8/EC, 1999/21/EC, 2006/125/EC and 2006/141/EC, Directive 2009/39/EC of the European Parliament and of the Council and Commission Regulations (EC) No 41/2009 and (EC) No 953/2009 Adopted by the Council on 22 April 2013#Statement by the Federal Republic of Germany#Statement by the United Kingdom | 52013AG0003 | 4/22/2013 | 6/15/2013 | Council of the European Union | |
| Regulation (EU) No 609/2013 of the European Parliament and of the Council of 12 June 2013 on food intended for infants and young children, food for special medical purposes, and total diet replacement for weight control and repealing Council Directive 92/52/EEC, Commission Directives 96/8/EC, 1999/21/EC, 2006/125/EC and 2006/141/EC, Directive 2009/39/EC of the European Parliament and of the Council and Commission Regulations (EC) No 41/2009 and (EC) No 953/2009 Text with EEA relevance | 32013R0609 | 6/12/2013 | 2014-05-29, 2013-06-29 | Council of the European Union, European Parliament | No end date |
| White Paper on a Strategy for Europe on Nutrition, Overweight and Obesity related health issues | 52007DC0279 | 5/30/2007 |  | European Commission | No end date |
| White Paper on Nutrition, Overweight and Obesity-related health issues European Parliament resolution of 25 September 2008 on the White Paper on nutrition, overweight and obesity-related health issues (2007/2285(INI)) | 52008IP0461 | 9/25/2008 | 1/14/2010 | Committee on the Internal Market and Consumer Protection, European Parliament, Committee on the Environment, Public Health and Food Safety, Committee on Women’s Rights and Gender Equality, Committee on Agriculture and Rural Development | |

REFERENCES

1. WHO NCD Management-Screening DaT. Global report on diabetes. France; 2016 21 April.

2. European Public Health Alliance. Joint Paper Towards an EU strategic framework for the prevention of NCDs. Brussels, Belgium: European Public Health Alliance; 2019.

3. Abiola SE, Mello MM. Multilevel legal approaches to obesity prevention: A conceptual and methodological toolkit. PLoS One. 2019;14(10):e0220971.
